# The Abuse of Cesarean Section

**Published:** 1888-12

**Authors:** 


### *THE ABUSE OF CESAREAN SECTION.*

At a recent meeting of the Philadelphia Obstetrical Society, a valuable paper was read by Dr. Joseph Price, of that city, on the above subject, a brief synopsis of which we find in the November number of the *American Journal of Obstetrics.* We refer to this subject with a view to endorse the principles enunciated by the writer. It is a noticeable fact that theories and practice fall into desuetude, or progress in favor, almost as a fashion. Until within a few years, the Cesarean section was performed only as a dernier resort, and was justified when the delivery of the fetus *per vias naturales* was impossible. A wise conservatism has prevailed in the profession in the position assumed on this important measure. Only "when the degree of contraction is one and a-half or two inches" was the accoucheur warranted in resorting to abdominal section. In cases of pelvic contraction, when diagnosticated prior to the full period of utero-gestation, the induction of premature labor gives a reasonable chance for the rescue of the child, while the risk to the mother is almost *nil.* The British rule, that Cesarean section should never be an operation of election, but one of necessity, furnishes the proper safeguard to the pregnant woman. Dr. Price well says that, "once establish the precedent that Cesarean section is an elective procedure in obstetrics," and thus by inference endorse the principle that abdominal section is justifiable even when premature labor at the seventh or eighth month of utero-gestation could have been performed, and the fashion of the day will have full swing, and the unfortunate patient, who may be suffering from minor pelvic contraction, will fall a victim to the knife of some youthful aspirant for professional fame, whose greed for notoriety will overcome all scruples for the safety of the valuable lives entrusted to his care.

These principles Dr. Price well illustrated by the report of two cases on which the justness of these important principles are based. We briefly epitomize the essential facts: A woman already delivered of a living child yet living at four years, followed by three other deliveries at full term with forceps in which all the children were dead. In the fifth pregnancy the Cesarean operation is decided upon. Passing into the care of another attendant, premature labor is induced, which results in a safe delivery of a child, whose head gives a bi-parietal diameter of three and one-fourth inches, the conjugate of the brim being three and a-half inches.

In the second case, the woman was in her third pregnancy, the first child having been delivered after thirty hours' labor with instruments, dying at birth. The second pregnancy, the child is delivered at full term without instruments, the child yet living. In the third pregnancy, the Cesarean section is performed, rescuing the child, and the mother recovered after a protracted convalescence.

These cases illustrate the tendency of the profession to resort to this operation simply because abdominal section is the fashion, the accoucheur losing sight of the fundamental principles which justify it, and of the alternative, which rescues the child and saves the mother without the danger of the major operation. It is this principle that Dr. Price boldly emphasizes, and which we fully endorse. It brings up the wise conservatism of the past, with the additional advantage of the progress made in abdominal section, which, if performed as a *necessity*, affords the patient the increased chance for recovery. Sänger's improved Cesarean section gives to mothers a larger percentage of recoveries than embryotomy, while the saving o child-life brings results which more than justifies the accoucheur in resorting to it in those cases of *necessity* which alone justifies its selection.

